# New Modes of Administering Iodine

**Published:** 1859-02

**Authors:** 


*New Modes of Administering Iodine.* — Efforts have lately been made in France to administer iodine in a more efficacious manner than had hitherto been done. Mr. Leriche, of Lyons, has published valuable articles in *L'Union Médicale*, wherein he endeavors to show that iodine, combined with vegetable substances, advantageously replaces cod-liver oil. He proposes a syrup made of the juice of water-cresses and iodine, and also an iodine wine. The syrup has the advantage of not fermenting, and contains exactly one grain of iodine per ounce. The wine is composed thus: Bordeaux wine eight ounces; concentrated infusion of red roses about thirteen drachms; tincture of iodine one drachm and a half. Eight ounces contain one grain of iodine. From one to six tablespoonfuls may be given daily, according to the indications and the age of patients. In the space of three years, M. Lerich treated 38 scrofulous patients with the wine; 21 were perfectly cured after a treatment steadily pursued for some time; 8 did not improve at all; and 9 improved but slightly, either because the treatment was carried on imperfectly, or because it was left off too soon.

M. Boinet, on the other hand, well known by long-continued investigations respecting the use of iodine, read, on the 28th of September last, before the Academy of Medicine of Paris, a paper, in which he proposes to use iodine as an article of food. The author administers iodine as found in nature, viz., combined with those plants which contain the greatest quantity of the alkaloid. The latter, being thus given in minute doses, in a continuous and almost imperceptible manner, yields most advantageous results. M. Boinet uses faci, marine plants, cruciferæ, salts containing iodine, and some mineral waters holding iodine in solution. His excipients are ordinary bread, ginger-bread, cakes, biscuits, chocolate, wine, beer, syrups, &c., some being especially calculated for children. Trials were begun by M. Boinet as far back as 1849, upon subjects suffering very severely from the various well-known scrofulous symptoms, and most of them were cured after continuing the iodized food for several months. The author has not found that iodine administered for a long time produced a loss of flesh and atrophy of certain organs. Far from having these effects, the iodine, in his hands, has invigorated patients, and favored the development of organs. Messrs. Chastin and Trousseau are to report upon the paper.—*The American Jour. Med. Sciences.*

